# Robot-Assisted Versus Conventional Harvesting of DIEP and Latissimus Dorsi Flaps for Breast Reconstruction in Post-Mastectomy Women: A Systematic Review and Meta-Analysis

**DOI:** 10.3390/jcm14030744

**Published:** 2025-01-24

**Authors:** Stiven Yusufov, Olesya Startseva, Sami Khalfaoui, Evgeniia Zhigailova, Mark Gabriyanchik, Dina Manasherova, Kakhaber Meskhi, Igor Reshetov

**Affiliations:** 1 Independent Researcher, Moscow 119991, Russia; dm3097@columbia.edu; 2 Department of Oncology, Radiotherapy and Reconstructive Surgery, N.V. Sklifosovsky Institute of Clinical Medicine, Sechenov First Moscow State Medical University (Sechenov University), Moscow 119991, Russia; startseva_o_i@staff.sechenov.ru (O.S.); khalfaoui_s@student.sechenov.ru (S.K.); zhigaylova_e_a@student.sechenov.ru (E.Z.); gabriyanchik_m_a@staff.sechenov.ru (M.G.); reshetov_i_v@staff.sechenov.ru (I.R.); 3 Plastic Surgery Department, N.V. Sklifosovsky Institute of Clinical Medicine, Sechenov First Moscow State Medical University (Sechenov University), Moscow 119991, Russia; meskhi_k_t@staff.sechenov.ru

**Keywords:** robotic-assisted surgery, breast reconstruction, surgical outcomes, flap

## Abstract

**Background/Objectives**: Robotic breast reconstruction is an innovative surgical technique that integrates robotic technology into breast reconstruction procedures, offering several advantages over conventional approaches. These benefits include enhanced visualization, increased surgical dexterity, and superior cosmetic outcomes. This study aims to comprehensively compare robotic-assisted and conventional breast reconstruction approaches in terms of complication profiles and operation-related measurements. **Methods**: A comprehensive search was conducted in PubMed, Embase, Scopus, Web of Science, Google Scholar, CENTRAL, and VHL from inception to October 2024 to identify relevant studies. Risk ratios for the following complications were calculated between the groups: donor site seroma, hematoma, infection, and unplanned reoperation. Mean differences were also calculated for the duration of surgery, length of postoperative hospital stays, and opioid use. **Results**: A meta-analysis was performed on 9 studies including a total of 1094 patients. No significant differences were found between the groups in the risk ratios for reoperation, seroma formation, delayed healing, infections, and hematomas. Similarly, there were no significant differences in postoperative opioid use. The duration of surgery was longer in the robot-assisted reconstruction group, whereas the duration of hospital stay was shorter compared to the conventional group. Meta-regression analysis for the duration of surgery model showed that none of the moderators had a statistically significant effect on this outcome. ROBINS-I assessment indicated that all the included studies had a serious risk of bias. **Conclusions**: Our results suggest that using a robot-assisted approach is associated with a shorter duration of hospital stay and a longer duration of surgery.

## 1. Introduction

Breast cancer is the most diagnosed cancer among women worldwide, with approximately 2.3 million new cases reported in 2022, accounting for about 12.5% of all new cancer diagnoses, according to the International Agency for Research on Cancer [1]. Early detection and effective treatment have significantly increased survival rates in developed regions, leading more women to seek reconstructive procedures following mastectomy. However, access to breast reconstruction varies notably depending on geographic location, healthcare infrastructure, cultural factors, and awareness [2]. This procedure is a vital component of breast cancer management, enhancing sexual well-being and self-confidence among survivors. Breast reconstruction is primarily categorized into implant-based and autologous-based methods.

Autologous breast reconstruction, while being more complex, typically yields superior aesthetic results and enhanced psychological benefits for patients. The Deep Inferior Epigastric Perforator (DIEP) flap technique utilizes the patient’s own abdominal tissue, preserving underlying muscle and minimizing postoperative abdominal weakness [3,4,5]. Despite this advantage, a significant percentage of patients still report weakness in the anterior abdominal wall, which may result from muscle denervation and nerve damage during dissection. The lower epigastric artery, located beneath the rectus abdominis, can be affected by trauma during surgery, leading to a 30% loss of muscle function one year postoperatively. The harvesting of the DIEP flap requires extensive dissection of the rectus abdominis fascia, which can extend up to 15 cm [6]. To mitigate these risks, minimally invasive endoscopic techniques and robot-assisted surgery can be employed, allowing for shorter incisions and improved vascular pedicle isolation. In cases where abdominal tissue is insufficient or additional support is required, the Latissimus Dorsi (LD) flap technique may be utilized, incorporating muscle, skin, and fat from the upper back, often in conjunction with breast implants to achieve the desired size and contour [7].

Robotic surgery was first utilized in 1985 for precise stereotactic neurosurgical biopsies, leading to its application in transurethral resection of the prostate. Subsequent iterations included robotic systems designed for prostatectomy. However, these early systems relied on fixed anatomical landmarks and were not well-suited for plastic surgery. New robotic technologies aimed to enhance surgical dynamics, with the Da Vinci system (Intuitive Surgical Inc., Sunnyvale, CA, USA) emerging as the dominant platform. This system incorporates advanced visualization technology, offering stable 3D-HD imaging of the surgical field and allowing surgeons autonomous control of an 8 mm endoscope. This newer camera design was significantly improved compared to previous bulky systems, providing clearer and brighter images of the surgical area [8,9].

Robotic breast reconstruction represents an innovative advancement in surgical techniques, integrating robotic technology into breast reconstruction procedures. This approach offers several benefits over traditional methods, including enhanced visualization, greater surgical dexterity, and improved cosmetic outcomes. Robotic-assisted surgery considerably improves the precision of DIEP and LD flaps’ harvesting, reducing tissue trauma. For DIEP flaps, robotic technology allows for the meticulous dissection of blood vessels and perforators, resulting in smaller, more precise incisions that reduce postoperative pain and expedite recovery. In LD flap reconstruction, robotic assistance facilitates careful dissection of the muscle while minimizing scarring.

Since the publication of the previous meta-analysis, new studies have been released [10]. Also, the previous meta-analysis raised a number of methodological issues: (1) the authors used, in one outcome, different studies by the same author from the same hospital and overlapping years of patient recruitment; (2) there were outcomes that were calculated by combining only two studies. These factors make this paper even more relevant despite the publication of the previous meta-analysis.

This study aimed to provide a comprehensive and updated analysis of the available evidence comparing the surgical outcomes of robot-assisted harvesting versus conventional harvesting of the DIEP flap and the LD flap for breast reconstruction in women following mastectomy.

## 2. Materials and Methods

This systematic review and meta-analysis were performed and reported in accordance with the Cochrane Collaboration Handbook for Systematic Review of Interventions and the Preferred Reporting Items for Systematic Reviews and Meta-Analyses (PRISMA) Statement guidelines [11,12]. The review was registered in the PROSPERO database (registration number: CRD42024610691).

### 2.1. Inclusion Criteria

Inclusion in this meta-analysis was restricted to studies that met all the following eligibility criteria: (1) randomized trials or non-randomized cohorts; (2) comparison of robotic flap harvesting with conventional methods; (3) the study was conducted on humans; and (4) published in English. Additionally, studies were included only if they reported any of the clinical outcomes of interest.

### 2.2. Search Strategy

We systematically searched PubMed, Embase, Scopus, Web of Science, Google Scholar, the Cochrane Central Register of Controlled Trials, and the Virtual Health Library from inception to October 2024 with the following search terms: ‘Robotic Breast Reconstruction’, ‘Conventional Breast Reconstruction’, ‘Autologous Breast Reconstruction’, ‘Minimally Invasive Reconstruction’, and ‘Patient-reported outcomes’. All search queries and results for each database are available in Table S1.

References from all included studies, previous systematic reviews, and meta-analyses were also searched manually for any additional studies. Two authors (S.Y. and E.Z.) independently extracted the data following predefined search criteria and quality assessment protocols.

### 2.3. Outcomes of Interest

Outcomes of interest included the development of donor site seroma, hematoma, infection, and unplanned reoperation. Additionally, the study assessed the duration of surgery, length of postoperative hospital stays, opioid use for pain management, and patient-reported outcomes using the BREAST-Q questionnaire.

### 2.4. Assessment of Heterogeneity

We evaluated the risk of bias with the Risk of Bias in Non-randomized Studies of Interventions tool (ROBINS-I) [12]. Two independent authors (S.Y. and S.K.) completed the risk of bias assessment. Disagreements were resolved through a consensus after discussing reasons for the discrepancy. Publication bias was investigated by funnel-plot analysis of point estimates in relation to study weights. To assess the robustness of our synthesized results, we performed a leave-one-out sensitivity analysis. Additionally, we conducted a meta-regression analysis to explore potential sources of heterogeneity.

### 2.5. Statistical Analysis

Risk ratios (RRs) with 95% confidence intervals (CIs) were used to compare treatment effects for categorical endpoints. Continuous outcomes were compared using mean differences (MDs). We assessed heterogeneity with I^2^ statistics; I^2^ values greater than 40% were considered significant for heterogeneity.

We used the DerSimonian and Laird random-effects model for outcomes with significant heterogeneity; for outcomes without significant heterogeneity, a fixed-effects model was employed. In addition, meta-regression analyses were conducted for outcomes with sufficient data and variability to explore potential sources of heterogeneity.

The analyses were performed using Review Manager 5.3 (Cochrane Center, Cochrane Collaboration, Copenhagen, Denmark), Python 3.7, and R 4.4.2 within the Google Colab environment.

## 3. Results

### 3.1. Baseline Characteristics of the Studies

**Figure 1:**
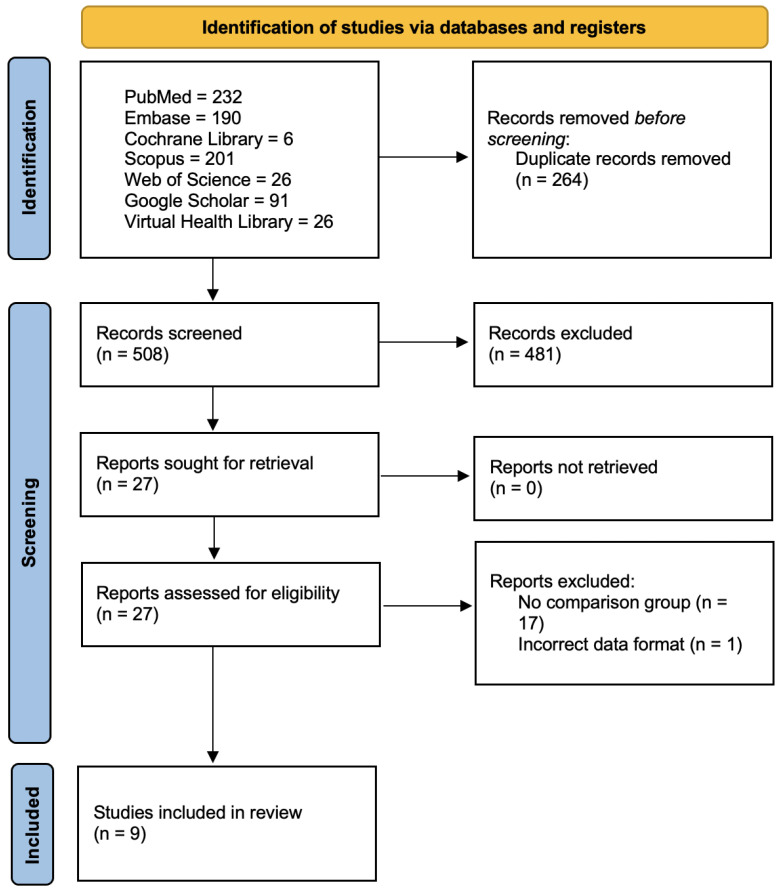
PRISMA 2020 flow diagram for systematic reviews and meta-analysis.

As seen in Figure 1, the initial search yielded 772 results. After the removal of duplicate records and ineligible studies, 27 articles remained and were fully reviewed based on the inclusion criteria. Of these, a total of 9 were eligible [13,14,15,16,17,18,19,20,21].

A total of 370 (33.8%) patients underwent robot-assisted reconstruction and 724 (66.2%) underwent conventional reconstruction. Study characteristics are summarized in Table 1. Five studies investigated the LD flap and four focused on the DIEP flap.

### 3.2. Analysis

**Figure 2:**
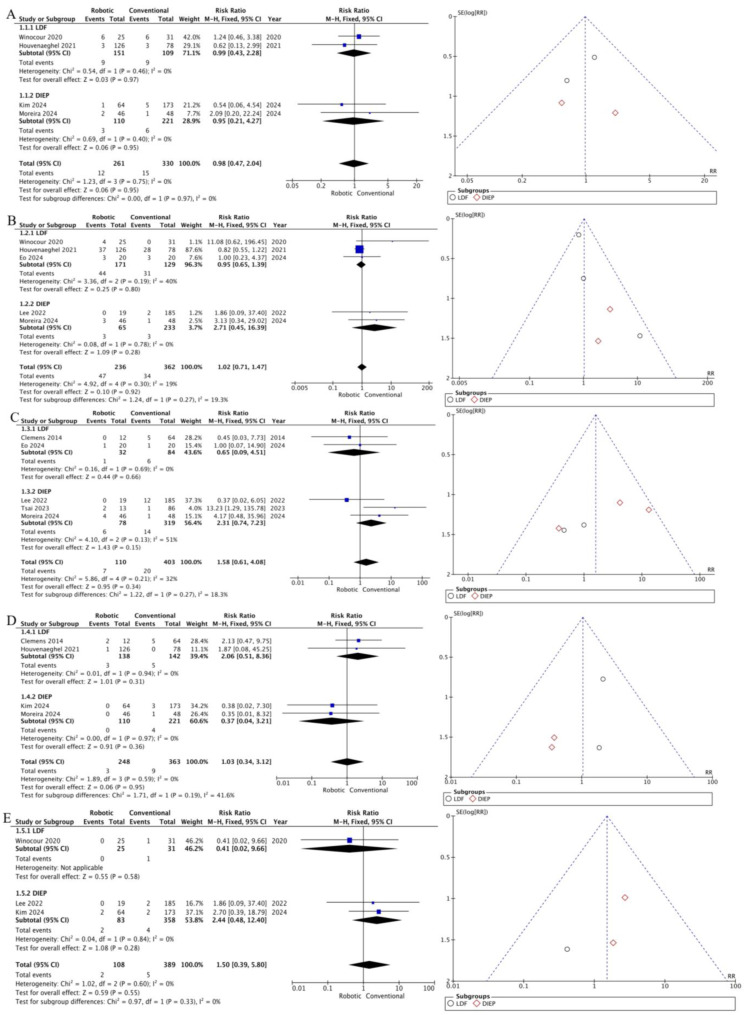
Forest plot of meta-analysis of the (**A**) reoperation, (**B**) donor-site seroma, (**C**) delayed healing, (**D**) donor-site infection, and (**E**) hematoma.

**Figure 3:**
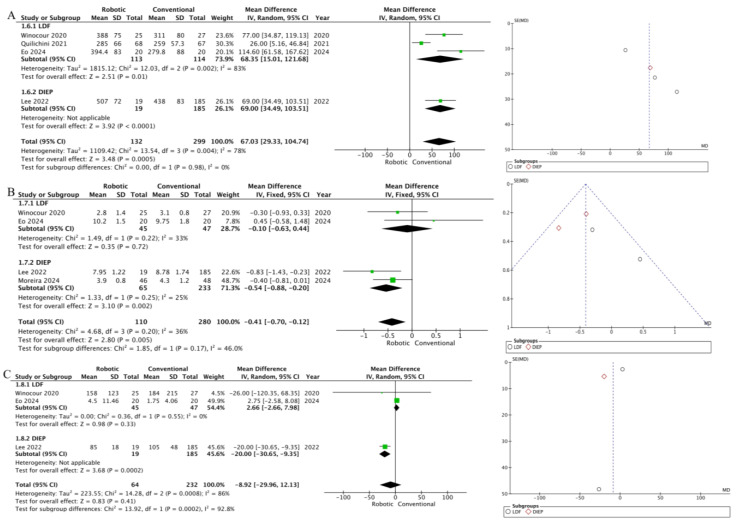
Forest plots of meta-analysis of the (**A**) duration of surgery (minutes), (**B**) postoperative stay (days), and (**C**) postoperative opioid use (morphine equivalent, mg).

Outcomes such as reoperation, seroma formation, delayed healing, infections, and hematomas were analyzed, along with the duration of surgery, postoperative hospital stay, and opioid use. Statistically significant differences were found in the analysis of operation duration and postoperative stay. The duration of surgery was longer in the group of robot-assisted reconstruction (MD 67.03; 95% CI [29.33; 104.74]; *p* = 0.0005, I^2^ = 78%). The duration of hospital stay was shorter in the robot-assisted group compared to the conventional group (MD −0.41; 95% CI [−0.70; −0.12]; *p* = 0.005; I^2^ = 36%). The analysis of the remaining outcomes did not show statistically significant differences, and the results are presented in Table 2 and Table 3 and Figure 2 and Figure 3.

### 3.3. Quality Assessment

ROBINS-I was used for quality assessment since all the studies included in the meta-analysis were not randomized. All studies were considered at serious risk of bias according to the ROBINS-I tool, as described in detail in Table S2.

In the funnel-plot analysis (Figure 2 and Figure 3) for outcomes such as reoperation, seromas, delayed healing, infections, hematomas, and postoperative hospital stay, the plots exhibited a symmetrical distribution as a function of weight and converged to a pooled effect as weight increased. In funnel-plot analyses (Figure 2 and Figure 3), for outcomes such as the duration of surgery and opioid use, the funnel plots showed greater heterogeneity and significant deviations from symmetry. This suggests that the results of these studies differ significantly from each other, and the pooled effect may be less reliable due to the high degree of heterogeneity between studies.

Leave-One-Out (LOO) sensitivity analysis was performed for all outcomes, except for the use of opiates, as only three studies were included in this outcome. The LOO analysis confirmed that excluding any single study from the analysis did not significantly change the overall results, which may indicate the validity and stability of the findings for these outcomes, and these results are presented in Figures S1 and S2.

Meta-regression was performed only for the duration of surgery, as this was the only statistically significant outcome with significant heterogeneity (I^2^ = 78%). Moderators such as BMI, mean age, percentage of patients with comorbidities, percentage of smokers, and percentage of patients with previous radiation therapy were included. The meta-regression model showed that none of these moderators had a statistically significant effect on the outcome. The results of the analysis are presented in Table S3 and Figure S3.

Meta-regression was used to assess the influence of moderators on the final effect measure. The main limitation of this analysis is the low numbers of studies. Three studies constitute an extremely small sample for a meta-regression, which strongly affects the statistical power and robustness of the obtained results. For the effect of age, we obtained the result of I^2^ = 0%, R^2^ = 100%, which tells us that all of the variation in outcomes can be defined by age. However, this result may be an artifact of the extremely small number of studies and the instability of the estimates. When the number of studies is very small, meta-regression models may produce unrealistically high or low estimates of heterogeneity, as well as odd R^2^ values. In practice, such a result should not be interpreted as “age explains everything” because the *p*-value is not significant, and the confidence intervals are wide. For the other moderators, I^2^ values were higher and R^2^ values were 0%, suggesting that these moderators do not explain much of the variability in the results.

## 4. Discussion

In this systematic review and meta-analysis of 9 studies and 1094 patients, we compared robotic-assisted to conventional breast reconstruction after mastectomy. The main findings of robotic surgery use include (1) shorter postoperative hospital stay and (2) longer duration of surgery.

We should consider our findings in terms of the existing prior meta-analyses on this topic [10]. Since the publication of the previous meta-analysis, new studies have been published, which increased the available evidence and encouraged us to reanalyze the available data. In addition, some methodological limitations were noted in that study, in particular, the use of multiple samples from the same hospital for the same endpoint in overlapping periods of patient recruitment, along with the calculation of some outcomes from two studies. Addressing these limitations was one of the motivations for conducting our analysis with a stricter approach to the selection and processing of primary data.

Robotic technologies have been less adopted in plastic surgery, but their use is gaining popularity in reconstructive and microsurgery. Regarding the increase in the duration of surgery, it is necessary to pay attention to the learning curve. Moreira et al. analyzed the DIEP pedicle dissection time curve as a function of the surgeon’s experience with the robot [22]. Their results were generally expected: dissection duration decreased over time, and it is also worth noting that peaks in the dissection duration were observed at 5–9 surgeries, which is associated with surgeons changing their technique to increase efficiency. It can be assumed that the further accumulation of experience by plastic surgeons is likely to help reduce the time required for the operation, making robot-assisted techniques more competitive. In this regard, it is clear that there is high heterogeneity (I^2^ = 78%) in this result, which is quite natural given that some surgeons have more experience compared to others. Moreover, some authors reported an increase in the overall duration of surgery due to the long preparation for the main stage. This includes the setup and calibration of the robotic system, positioning of the patient, etc. [23,24]. Nevertheless, with ongoing surgeon training and improvements in robotic technology, the required additional time for robot-assisted procedures is expected to decrease.

Our results showed a statistically significant reduction in the number of days of hospital stay after surgery using robot-assisted reconstruction. Although the results did not show significant heterogeneity, it is worth emphasizing the large variation between studies, which is due to the differences in clinical protocols between different countries and institutions. In addition, the minimally invasive nature of robot-assisted procedures may lead to a reduction in postoperative pain, allowing patients to be discharged earlier [25]. Even though our results showed no statistically significant differences in the use of opioid analgesics (the data were converted to morphine equivalent for calculation), there was a high level of heterogeneity, which may also be due to differences in the protocol for the use of this kind of anesthesia. Overall, the shorter postoperative hospitalization observed with robotic technology already highlights its potential to improve surgical efficiency and patient outcomes in reconstructive surgery.

Some authors have hypothesized that robot-assisted flap harvesting may reduce postoperative complications. This assumption is based on the idea of robotic technologies providing high-definition three-dimensional imaging, eliminating tremors, and providing seven degrees of freedom, which improves limb dexterity [26,27]. Our results are in contrast to this assumption, and specifically, the results of a meta-analysis of studies comparing the number of complications in the robot-assisted and conventional surgery groups showed no statistically significant differences between the groups in the following outcomes: reoperations, donor seromas, delayed healing, development of infections, and hematomas. These results may also be related to the learning curve. In their paper, Houvenaeghel et al. divided patients into two groups: the first group consisted of patients who underwent reconstruction in 2016–2017 and the second consisted of those who had surgery in 2018–2020 [15]. Their data showed a decrease in the number of complications in absolute and relative numbers in the second group. However, these data cannot be fully implemented to the results we obtained because in the study of Houvenaeghel et al., patients were not divided into robot-assisted reconstruction and conventional reconstruction groups, and their results were not statistically significant [15]. The data suggest that while robotic techniques theoretically offer advantages in surgical precision and control, those advantages may not yet be capable of leading to a measurable reduction in postoperative complications. It is not inconceivable that as surgeons become more proficient with robotic systems and technology advances, both theoretical and clinical differences may appear.

The BREAST-Q is the only widely used patient-reported outcome measure in questionnaire format to assess the quality of life and patient satisfaction related to breast surgery [28]. In this study, we would like to analyze the BREAST-Q score, but only Lee et al. and Kim et al. reported BREST-Q results [18,19]. However, in Kim et al.’s study, whereby they analyzed BREAST-Q scores by comparing a conventional group of patients with robot-assisted flap-based and implant-based breast reconstruction, only the overall completion rate was shown [18]. Consequently, even an analysis with a small number of studies was not possible because the number of participating patients from each group was not presented. Therefore, the inability to aggregate the BREAST-Q data shows a significant gap in the current literature, emphasizing the need for future studies.

Nevertheless, it is important to note that this study has significant limitations. First, the level of evidence is not high enough, as all studies included in the meta-analysis were retrospective and prospective cohort studies. To improve the accuracy of the meta-analysis, it is recommended that better-quality randomized controlled trials be included when they become available. Future studies should aim to standardize postoperative management strategies. Second, a significant limitation of our meta-analysis is the wide heterogeneity of patient- and surgical technique-related characteristics. In this regard, it is particularly notable that there are no generally accepted protocols for robot-assisted flap harvesting for breast reconstruction. The techniques described by the authors for robot-assisted DIEP flap harvesting are aimed at maximally reducing damage to the anterior abdominal wall, improving visualization of the vascular pedicle, and reducing scarring. The main differences between the techniques concern access (incision size and location, port placement) and intra-abdominal dissection features (working pressure and sequence) [19,20,21]. Also, a variety of robotic techniques for harvesting the LD flap have been reported in the literature, which differs in patient position, incision length and location, and the method of port placement [14,15,17]. All these differences in detail contribute to the increased heterogeneity of the combined result.

The development of robot-assisted plastic surgery relies on future improvements in surgeons’ skills and robot design. In addition, whether robot-assisted surgery is the approach of choice will depend on various factors that include cost, institutional resources, and the learning curve required to achieve mastery. Although robotic-assisted procedures show the potential to reduce hospital stays, they require a larger investment and a longer training period. With more centers adopting robotic platforms and accumulating long-term data, robust comparative studies will be key to clarifying the role and value of robotic surgery in reconstructive and aesthetic interventions.

Improving training programs to decrease the learning curve and optimizing robotic technology could potentially reveal the full clinical benefits of robotic surgery, leading to more successful patient outcomes in the future. Multicenter randomized controlled trials with defined procedure protocols are needed to draw clearer conclusions.

## 5. Conclusions

Our findings indicate that using a robot-assisted approach is associated with a shorter duration of hospital stay and a longer duration of surgery. Future research should focus on high-quality randomized controlled trials, the standardization of surgical and postoperative protocols, and the impact of surgeon experience in order to fully exploit the potential benefits of robot-assisted surgery.

## Figures and Tables

**Table 1:** Baseline characteristics of included studies.

Study	Design	Flap	State	Periods	Patients	Average Age, Year	Previous Radiation, %	Previous Chemotherapy, %	Average BMI	Comorbidities, %	Smokers, %	Average Follow-Up, mo
Clemens 2014 [13]	Retrospective	LDF	Houston, Texas	2009–2013	12/64	54.3/56.1	100/100	NA	25.4/25.9	16.6/18.8	25/21.9	12.3/16.4
Winocour 2020 [17]	Retrospective	LDF	Houston, Texas	2011–2015	25/25	51 (9.7)/50 (8.7)	72/59	72/67	24 (3.2)/29.8 (6.1)	0/30	0/4	60/12
Houvenaeghel 2021 [15]	Prospective	LDF	Marseille, France	2016–2020	126/78	54.5 (52.94–57.44)/50.5 (47.53–53.06)	42.1/62.8	NA	23.51 (24.04−25.69)/23.7 (23.41−25.06)	NA	27/17.9	NA
Quilichini 2021 [16]	Retrospective	LDF	Marseille, France	2016–2019	68/67	NA	NA	NA	NA	NA	NA	NA
Lee 2022 [19]	Retrospective	DIEP	Seoul, South Korea	2017–2021	19/185	47.8 (5.7)/48.6 (7.9)	NA	21/16	23.6 (3.5)/24.0 (3.1)	5/17	0/2	NA
Tsai 2023 [21]	Retrospective	DIEP	Zurich, Switzerland	2020–2022	13/86	46 (10.96)/45.6 (7.24)	NA	NA	23.5 (2.95)/24.4 (3.59)	15.4/4.6	0/1.2	15.0 (9.3)/14.0 (7.3)
Eo 2024 [14]	Prospective	LDF	Daegu, Korea	2020–2021	20/20	45.4 (5.7)/46.6 (4.8)	100/100	65/55	23.7 (3.3)/22.8 (2.7)	0/5	0/0	18.4 (4.6)/18.4 (7.1)
Kim 2024 [18]	Retrospective	DIEP	Seoul, Republic of Korea	2019–2021	64/173	NA	NA	NA	NA	NA	NA	NA
Moreira 2024 [20]	Retrospective	DIEP	Pittsburgh, Pennsylvania	2021–2022	23/24	49.3 (10.7)/49.4 (10.5)	34.8/33.3	52.2/45.8	29.5 (5.1)/29.0 (4.7)	NA	0/0	9.3/11.9

**Table 2:** The results of a meta-analysis of studies examining complications.

Outcome	Group	Events, % (R-a vs. Con.)	Risk Ratio	95% CI	*p*-Value	I^2^, %
Reoperation	LDF	6.1 vs. 9.8	0.92	0.42 to 1.99	0.82	0
	DIEP	2.7 vs. 2.7	0.95	0.21 to 4.27	0.95	0
	Total	4.8 vs. 5.8	0.93	0.46 to 1.84	0.82	0
Seroma	LDF	24.6 vs. 19.7	0.94	0.65 to 1.37	0.76	10
	DIEP	4.6 vs. 1.3	2.71	0.45 to 16.39	0.28	0
	Total	19.4 vs. 9.62	1.00	0.70 to 1.44	0.98	0
Delayed healing	LDF	3.1 vs. 7.1	0.65	0.09 to 4.51	0.66	0
	DIEP	7.7 vs. 4.4	2.31	0.74 to 7.23	0.15	51
	Total	6.4 vs. 5	1.58	0.61 to 4.08	0.34	32
Infection	LDF	2.2 vs. 3.5	2.06	0.51 to 8.36	0.31	0
	DIEP	0 vs. 1.8	0.37	0.04 to 3.21	0.36	0
	Total	1.2 vs. 2.5	1.03	0.34 to 3.12	0.95	0
Haematoma	DIEP	2.4 vs. 1.1	2.44	0.48 to 12.40	0.28	0
	Total	1.9 vs. 1.3	1.50	0.39 to 5.80	0.55	0

**Table 3:** The results of a meta-analysis of studies examining operation-related factors.

Outcome	Group	Mean Difference	95% CI	*p*-Value	I^2^, %
Duration of surgery	LDF	68.35	15.01 to 121.68	0.01	83
	Total	67.03	29.33 to 104.74	0.0005	78
Postoperative stay	LDF	−0.10	−0.63 to 0.44	0.72	33
	DIEP	−0.54	−0.88 to −0.20	0.002	25
	Total	−0.41	−0.70 to −0.12	0.005	36
Opiate using	LDF	2.66	−2.66 to 7.98	0.33	0
	Total	−8.92	−29.96 to 12.13	0.41	86

## Supplementary Materials

The following supporting information can be downloaded at: https://www.mdpi.com/article/10.3390/jcm14030744/s1, Figure S1: Leave-One-Out analysis; Figure S2: Leave-One-Out analysis; Figure S3: Meta-regression plots for each potential moderator; Table S1: Search queries and results for each database; Table S2: ROBINS-I bias assessment results; Table S3: Results of meta-regression analyses for potential moderators.

## Abbreviations

The following abbreviations are used in this manuscript:

CI	Confidence interval
DIEP	Deep Inferior Epigastric Perforator
LD	Latissimus Dorsi
LOO	Leave-One-Out
MD	Mean difference
PRISMA	Preferred Reporting Items for Systematic Reviews and Meta-Analyses
ROBINS-I	Risk of Bias in Non-randomized Studies of Interventions tool
RR	Risk ratio
