# Supplementary material for: Robot-Assisted Versus Conventional Harvesting of DIEP and Latissimus Dorsi Flaps for Breast Reconstruction in Post-Mastectomy Women: A Systematic Review and Meta-Analysis
Source: J Clin Med. 2025 Jan 24;14(3):744. doi: 10.3390/jcm14030744 (PMC11818507; doi:10.3390/jcm14030744)
Supplement: Supplementary file 1 [file jcm-14-00744-s001.zip › jcm-3426152-supplementary.pdf]

Database	Search query	Result	Date
PubMed	(post oncology mastectomy OR mastectomy) AND (robotic breast reconstruction OR robotic-assisted reconstruction) AND (classic breast reconstruction OR small invasive reconstruction OR minimally invasive reconstruction) AND (surgical outcomes OR BreastQ OR patient-reported outcomes OR PROs)	23	06.11.2024
	(post oncology mastectomy OR breast cancer mastectomy OR mastectomy) AND (robotic breast reconstruction OR robot-assisted breast reconstruction OR robotic-assisted reconstruction) AND (classic breast reconstruction OR conventional breast reconstruction OR minimally invasive reconstruction OR small incision reconstruction) AND (surgical outcomes OR BreastQ OR patient-reported outcomes OR quality of life OR complications OR PROs)	54	06.11.2024
	(Robot-assisted breast reconstruction OR Robotic breast reconstruction OR Robotic-assisted mastectomy reconstruction OR Robotic surgery breast reconstruction OR Minimally invasive robotic breast reconstruction OR Robotic flap reconstruction OR Robotic DIEP flap reconstruction OR Robotic nipple-sparing mastectomy reconstruction OR Robotic-assisted tissue reconstruction OR Robotic-assisted autologous breast reconstruction OR Robotic mastectomy with reconstruction OR Robotic-assisted free flap breast reconstruction OR Robotic TRAM flap reconstruction OR robotic) AND (Traditional breast reconstruction OR Conventional breast reconstruction OR Open breast reconstruction OR Standard breast reconstruction OR Classic flap breast reconstruction OR Small incision breast reconstruction OR Minimally invasive breast reconstruction OR Less invasive breast reconstruction OR Conventional surgical breast reconstruction OR Open approach breast reconstruction OR Reduced scar breast reconstruction OR Limited incision breast reconstruction OR Classic surgical breast reconstruction OR Traditional mastectomy reconstruction OR Small cut breast reconstruction OR Autologous Breast Reconstruction) (Robot-assisted breast reconstruction OR Robotic breast reconstruction OR Robotic-assisted mastectomy reconstruction OR Robotic surgery breast reconstruction OR Minimally invasive robotic breast reconstruction OR Robotic flap reconstruction OR Robotic DIEP flap reconstruction OR Robotic nipple-sparing mastectomy reconstruction OR Robotic-assisted tissue reconstruction OR Robotic-assisted autologous breast reconstruction OR Robotic mastectomy with reconstruction OR Robotic-assisted free flap breast reconstruction OR Robotic TRAM flap reconstruction OR robotic) AND (Traditional breast reconstruction OR Conventional breast reconstruction OR Open breast reconstruction OR Standard breast reconstruction OR Classic flap breast reconstruction OR Small incision breast reconstruction OR Minimally invasive breast reconstruction OR Less invasive breast reconstruction OR Conventional surgical breast reconstruction OR Open approach breast reconstruction OR Reduced scar breast reconstruction OR Limited incision breast reconstruction OR Classic surgical breast reconstruction OR Traditional mastectomy reconstruction OR Small cut breast reconstruction OR Autologous Breast Reconstruction)	155	06.11.2024
Embase	(Robot-assisted breast reconstruction OR Robotic breast reconstruction OR Robotic-assisted mastectomy reconstruction OR Robotic surgery breast reconstruction OR Minimally invasive robotic breast reconstruction OR Robotic flap reconstruction OR Robotic DIEP flap reconstruction OR Robotic nipple-sparing mastectomy reconstruction OR Robotic-assisted tissue reconstruction OR Robotic-assisted autologous breast reconstruction OR Robotic mastectomy with reconstruction OR Robotic-assisted free flap breast reconstruction OR Robotic TRAM flap reconstruction OR robotic) AND (Traditional breast reconstruction OR Conventional breast reconstruction OR Open breast reconstruction OR Standard breast reconstruction OR Classic flap breast reconstruction OR Small incision breast reconstruction OR Minimally invasive breast reconstruction OR Less invasive breast reconstruction OR Conventional surgical breast reconstruction OR Open approach breast reconstruction OR Reduced scar breast reconstruction OR Limited incision breast reconstruction OR Classic surgical breast reconstruction OR Traditional mastectomy reconstruction OR Small cut breast reconstruction OR Autologous Breast Reconstruction)	190	06.11.2024
Cochrane Library	(Robot-assisted breast reconstruction OR Robotic breast reconstruction OR Robotic-assisted mastectomy reconstruction OR Robotic surgery breast reconstruction OR Minimally invasive robotic breast reconstruction OR Robotic flap reconstruction OR Robotic DIEP flap reconstruction OR Robotic nipple-sparing mastectomy reconstruction OR Robotic-assisted tissue reconstruction OR Robotic-assisted autologous breast	6	06.11.2024

	reconstruction OR Robotic mastectomy with reconstruction OR Robotic-assisted free flap breast reconstruction OR Robotic TRAM flap reconstruction OR robotic) AND (Traditional breast reconstruction OR Conventional breast reconstruction OR Open breast reconstruction OR Standard breast reconstruction OR Classic flap breast reconstruction OR Small incision breast reconstruction OR Minimally invasive breast reconstruction OR Less invasive breast reconstruction OR Conventional surgical breast reconstruction OR Open approach breast reconstruction OR Reduced scar breast reconstruction OR Limited incision breast reconstruction OR Classic surgical breast reconstruction OR Traditional mastectomy reconstruction OR Small cut breast reconstruction OR Autologous Breast Reconstruction)		
Scopus	ALL ((("Robot-assisted breast reconstruction") OR ("Robotic breast reconstruction") OR ("Robotic-assisted mastectomy reconstruction") OR ("Robotic surgery breast reconstruction") OR ("Minimally invasive robotic breast reconstruction") OR ("Robotic flap reconstruction") OR ("Robotic DIEP flap reconstruction") OR ("Robotic nipple-sparing mastectomy reconstruction") OR ("Robotic-assisted tissue reconstruction") OR ("Robotic-assisted autologous breast reconstruction") OR ("Robotic mastectomy with reconstruction") OR ("Robotic-assisted free flap breast reconstruction") OR ("Robotic TRAM flap reconstruction") OR (robotic)) AND (("Traditional breast reconstruction") OR ("Conventional breast reconstruction") OR ("Open breast reconstruction") OR ("Standard breast reconstruction") OR ("Classic flap breast reconstruction") OR ("Small incision breast reconstruction") OR ("Minimally invasive breast reconstruction") OR ("Less invasive breast reconstruction") OR ("Conventional surgical breast reconstruction") OR ("Open approach breast reconstruction") OR ("Reduced scar breast reconstruction") OR ("Limited incision breast reconstruction") OR ("Classic surgical breast reconstruction") OR ("Traditional mastectomy reconstruction") OR ("Small cut breast reconstruction") OR ("Autologous Breast Reconstruction")))	201	06.11.2024
Web of Science	(("Robot-assisted breast reconstruction") OR ("Robotic breast reconstruction") OR ("Robotic-assisted mastectomy reconstruction") OR ("Robotic surgery breast reconstruction") OR ("Minimally invasive robotic breast reconstruction") OR ("Robotic flap reconstruction") OR ("Robotic DIEP flap reconstruction") OR ("Robotic nipple-sparing mastectomy reconstruction") OR ("Robotic-assisted tissue reconstruction") OR ("Robotic-assisted autologous breast reconstruction") OR ("Robotic mastectomy with reconstruction") OR ("Robotic-assisted free flap breast reconstruction") OR ("Robotic TRAM flap reconstruction") OR (robotic)) AND (("Traditional breast reconstruction") OR ("Conventional breast reconstruction") OR ("Open breast reconstruction") OR ("Standard breast reconstruction") OR ("Classic flap breast reconstruction") OR ("Small incision breast reconstruction") OR ("Minimally invasive breast reconstruction") OR ("Less invasive breast reconstruction") OR ("Conventional surgical breast reconstruction") OR ("Open approach breast reconstruction") OR ("Reduced scar breast reconstruction") OR ("Limited incision breast reconstruction") OR ("Classic surgical breast reconstruction") OR ("Traditional mastectomy reconstruction") OR ("Small cut breast reconstruction") OR ("Autologous Breast Reconstruction"))	26	06.11.2024
Google Scholar	(Robot-assisted breast reconstruction OR Robotic breast reconstruction OR Robotic-assisted mastectomy reconstruction OR Robotic surgery breast reconstruction OR Minimally invasive robotic breast reconstruction OR Robotic flap reconstruction OR Robotic DIEP flap reconstruction OR Robotic nipple-sparing mastectomy reconstruction OR Robotic-assisted tissue reconstruction OR Robotic-assisted autologous breast reconstruction OR Robotic mastectomy with reconstruction OR Robotic-assisted free flap breast reconstruction OR Robotic TRAM flap reconstruction OR robotic) AND (Traditional breast reconstruction OR Conventional breast reconstruction OR Open breast reconstruction OR Standard breast reconstruction OR Classic flap breast reconstruction OR Small incision breast reconstruction OR Minimally invasive breast reconstruction OR Less invasive breast reconstruction OR Conventional surgical breast reconstruction OR Open approach breast reconstruction OR Reduced scar breast reconstruction OR Limited incision breast reconstruction OR Classic surgical breast reconstruction OR Traditional mastectomy reconstruction OR Small cut breast reconstruction OR Autologous Breast Reconstruction)	91	06.11.2024
Virtual Health Library	(("Robot-assisted breast reconstruction") OR ("Robotic breast reconstruction") OR ("Robotic-assisted mastectomy reconstruction") OR ("Robotic surgery breast reconstruction") OR ("Minimally invasive robotic breast reconstruction") OR ("Robotic flap reconstruction") OR ("Robotic DIEP flap reconstruction") OR ("Robotic nipple-sparing mastectomy reconstruction") OR ("Robotic-assisted tissue reconstruction")	26	06.11.2024

	OR ("Robotic-assisted autologous breast reconstruction") OR ("Robotic mastectomy with reconstruction") OR ("Robotic-assisted free flap breast reconstruction") OR ("Robotic TRAM flap reconstruction") OR (robotic)) AND (("Traditional breast reconstruction") OR ("Conventional breast reconstruction") OR ("Open breast reconstruction") OR ("Standard breast reconstruction") OR ("Classic flap breast reconstruction") OR ("Small incision breast reconstruction") OR ("Minimally invasive breast reconstruction") OR ("Less invasive breast reconstruction") OR ("Conventional surgical breast reconstruction") OR ("Open approach breast reconstruction") OR ("Reduced scar breast reconstruction") OR ("Limited incision breast reconstruction") OR ("Classic surgical breast reconstruction") OR ("Traditional mastectomy reconstruction") OR ("Small cut breast reconstruction") OR ("Autologous Breast Reconstruction"))		

Table S1. Search queries and results for each database.

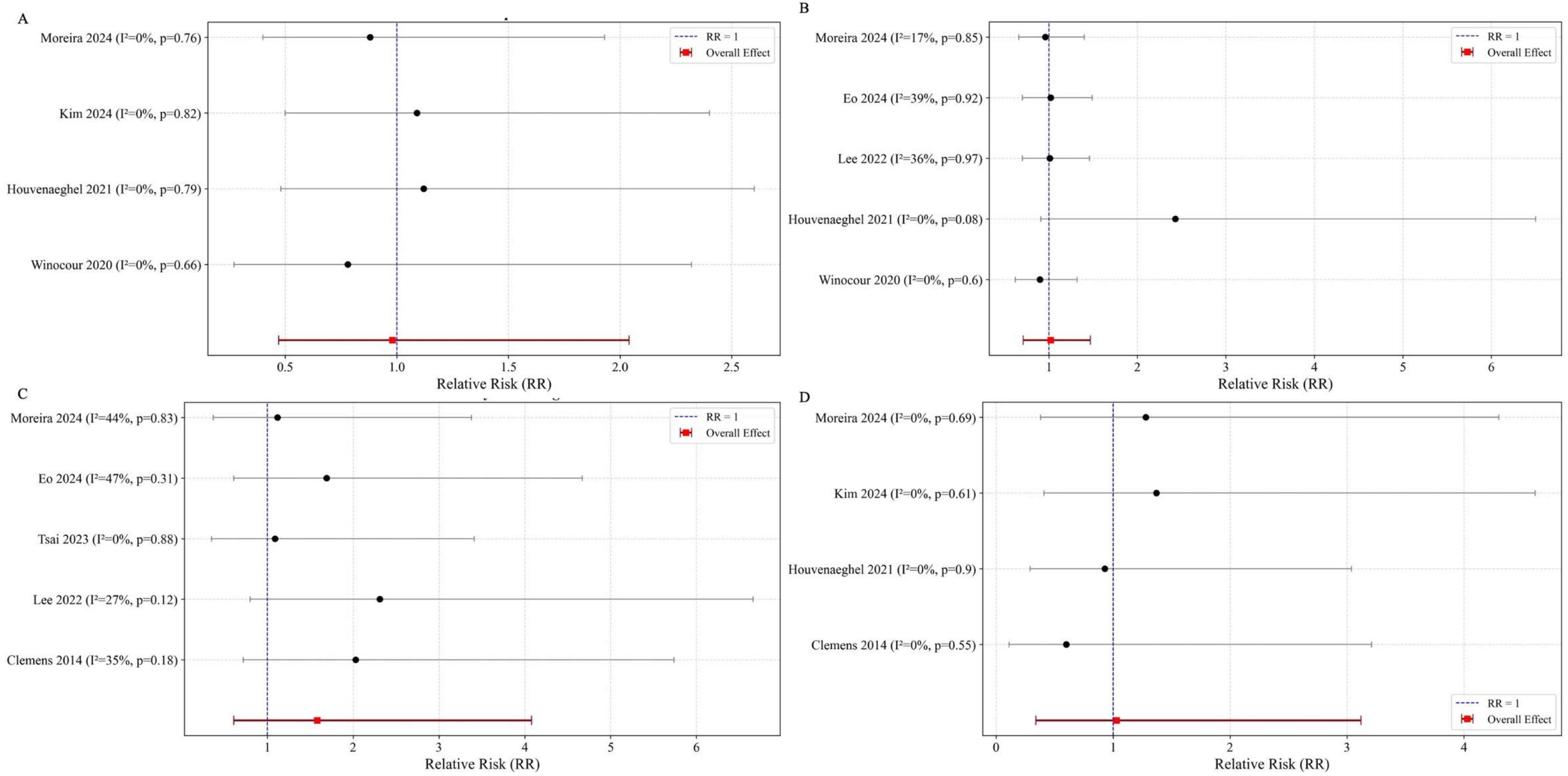

Figure S1. Leave-One-Out analysis. A – reoperation rate, B – donor-site seroma, C – delyaed healing, D – infection.

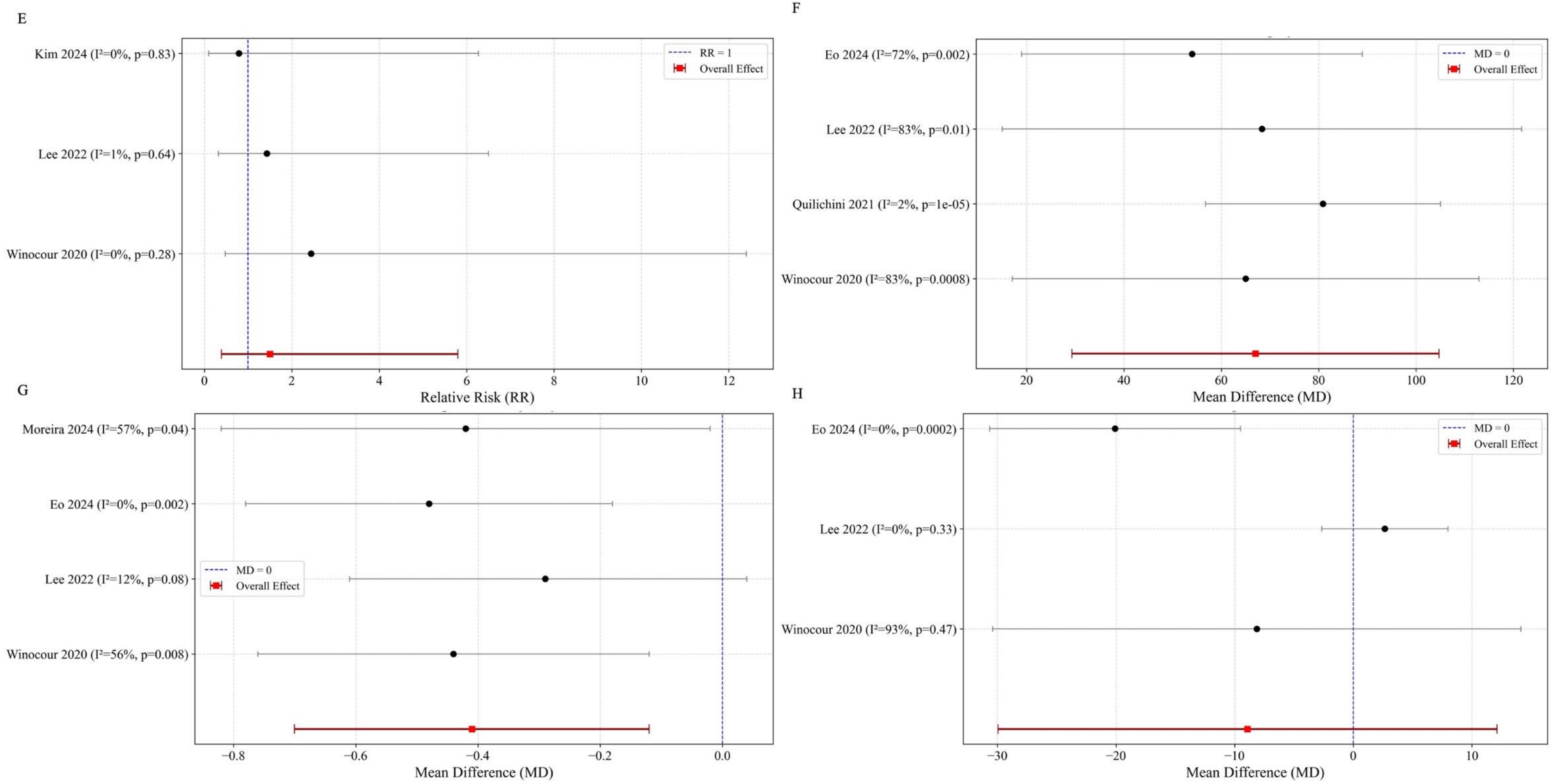

Figure S2. Leave-One-Out analysis. A- donor-site hematoma, B – duration of surgery, C – post operative stay, D - opiate using.

Study	Bias due to confounding	Bias in selection of participants into the study	Bias in classification of interventions	Bias due to deviations from intended interventions	Bias due to missing data	Bias in measurement of outcomes	Bias in selection of the reported result	Overall Bias
Clemens, 2014	Serious	Low	Low	Moderate	Low	Serious	Low	Serious
Winocour, 2020	Serious	Moderate	Low	Moderate	Low	Serious	Low	Serious
Houvenaeghel, 2021	Serious	Moderate	Low	Moderate	Low	Serious	Low	Serious
Quilichini, 2021	Serious	Moderate	Low	Moderate	Moderate	Serious	Low	Serious
Lee, 2022	Moderate	Moderate	Low	Moderate	Low	Serious	Low	Serious
Tsai, 2023	Serious	Moderate	Low	Moderate	Low	Serious	Low	Serious
Eo, 2024	Serious	Moderate	Low	Moderate	Low	Serious	Low	Serious
Kim, 2024	Serious	Moderate	Low	Moderate	Low	Serious	Low	Serious
Moreira, 2024	Serious	Moderate	Low	Moderate	Low	Serious	Low	Serious

Table S2. ROBINS-I bias assesment results.

Figure S3. Meta-Regression Plots for Each Potential Moderator. A – comorbidities, B – smokers, C – BMI, D – Previous radiation, E – mean age.

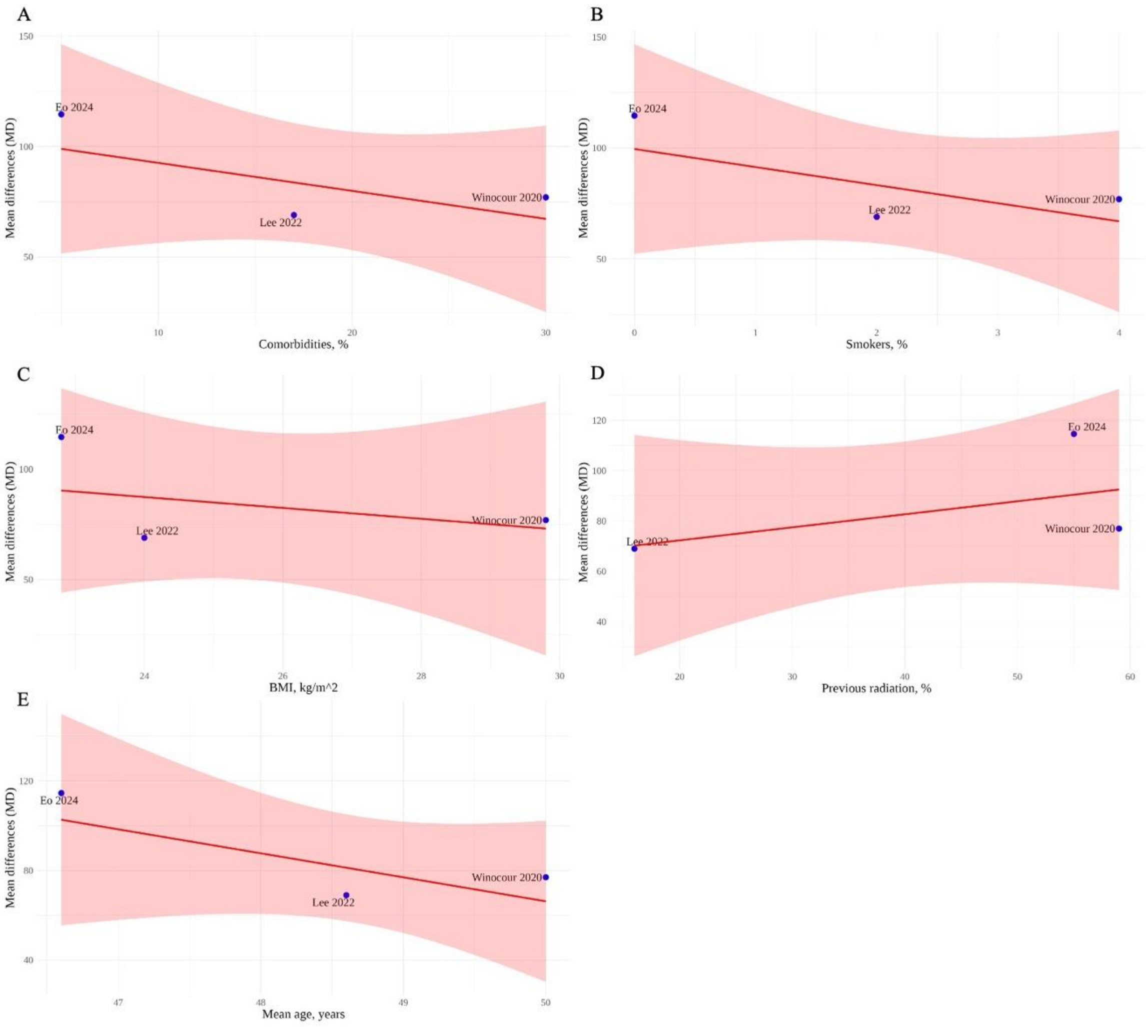

Table S3. Results of Meta-Regression Analyses for Potential Moderators

Moderator	Moderator Estimate	Moderator p-value	95% CI Lower	95% CI Upper	I^2 (%)	R^2 (%)	QE (df=1)	QE p-value	QM (df=1)	QM p-value
Age	-10.72 (10.16)	0.2914	-30.62	9.19	0.00	100.00	0.9284	0.3353	1.1132	0.2914
Previous Radiation	0.52 (0.72)	0.4708	-0.89	1.93	24.20	0.00	1.92	0.2507	0.5202	0.4708
BMI	-2.46 (5.72)	0.6671	-13.67	8.75	46.12	0.00	1.61	0.1731	0.1851	0.6671
Comorbidities	-1.27 (1.47)	0.3886	-4.15	1.61	19.07	0.00	1.57	0.2663	0.7432	0.3886
Smokers	-8.14 (9.07)	0.3691	-25.91	9.63	15.28	0.00	1.1804	0.2773	0.8067	0.3691
